# A Case of Atypical Hairy Cell Leukemia With CD10+ and CD38+: Diagnosis and Treatment

**DOI:** 10.7759/cureus.31882

**Published:** 2022-11-25

**Authors:** Tung Tuan Nguyen, An Thi Vinh Do, Phuong Phan Thi, Le Lan Anh, Minh Tam Vu

**Affiliations:** 1 Hematology and Blood Transfusion, Bach Mai Hospital, Hanoi, VNM

**Keywords:** hematology, cd38, cd10, lymphoid neoplasm, hairy cell leukemia

## Abstract

Hairy cell leukemia (HCL) is a rare disease of mature B-cell neoplasms. Its name comes from the hair-like strands surrounding the cytoplasm of the cells, which are observed on peripheral blood or bone marrow smears. Leukemic cells mainly involve the spleen, peripheral blood, and bone marrow. The classical immunophenotyping of HCL includes overexpression of the B-cell surface antigens such as CD19, CD20, and CD22 and co-expression of CD25, CD103, CD11c, and CD123. Other markers including CD5, CD10, and CD38 are usually negative, in which CD38 is considered a poor prognostic factor. Herein, we report a case of HCL with atypical morphology and abnormal expression of both CD38 and CD10.

## Introduction

Originally described in 1958 [1], hairy cell leukemia (HCL) is a disease that can be clearly and distinctly defined in the 2008 revision of the World Health Organization (WHO) classification of hematopoietic and lymphoid tumors [2]. With over 1,000 new cases annually in Europe and the United States, HCL is more common in men with an average age of about 60 years [3,4]. The disease progresses indolently with no specific cause, and patients often present with symptoms of splenomegaly, thrombocytopenia, or infection. Peripheral lymphadenopathy is rarely seen in most cases. Definitive diagnosis is based on the presence of hairy cells in the peripheral blood or bone marrow and its diffuse infiltrates in the endothelial system such as the spleen [5].

HCL is a rare, chronic B-cell malignancy that accounts for about 2% of lymphoid neoplasms [2]. It can be confused with other B-cell disorders, including splenic marginal zone lymphoma (SMZL). HCL is characterized by lymphocytes with ovoid nuclei and abundant and pale hair-like cytoplasm. While SMZL is characterized by the presence of villus lymphocytes with round nuclei, dense chromatin, and basophilic cytoplasm with short polarized villi in the peripheral blood. To differentiate these diseases, flow cytometry was used and a scale was constructed with four characteristic markers (CD11c, CD25, HC2, and B-ly-7 (CD103)): one point when it is expressed and no point when it is not expressed. Of the cases of HCL, 98% have a score of 3 or 4, whereas a lower score is in HCL-like disorders [6]. CD200 with clear expression may be useful for the diagnosis of HCL [7]. CD5, CD10, and CD38 are usually negative; 10-18% of cases of HCL have occasionally been reported with CD10 expression, meanwhile, CD38 has appeared at a higher rate in studies (20-30%) [2].

Chemotherapy is the main treatment for HCL with single-drug treatment regularly. Rituximab, a monoclonal antibody, can sometimes be used in combination with chemotherapy. The spleen can reduce in size after chemotherapy. Therefore, splenectomy is rarely used in patients with HCL, and it should only be performed on patients who have clinical discomfort or decreased blood cell count [2].

## Case presentation

**Figure 1:**
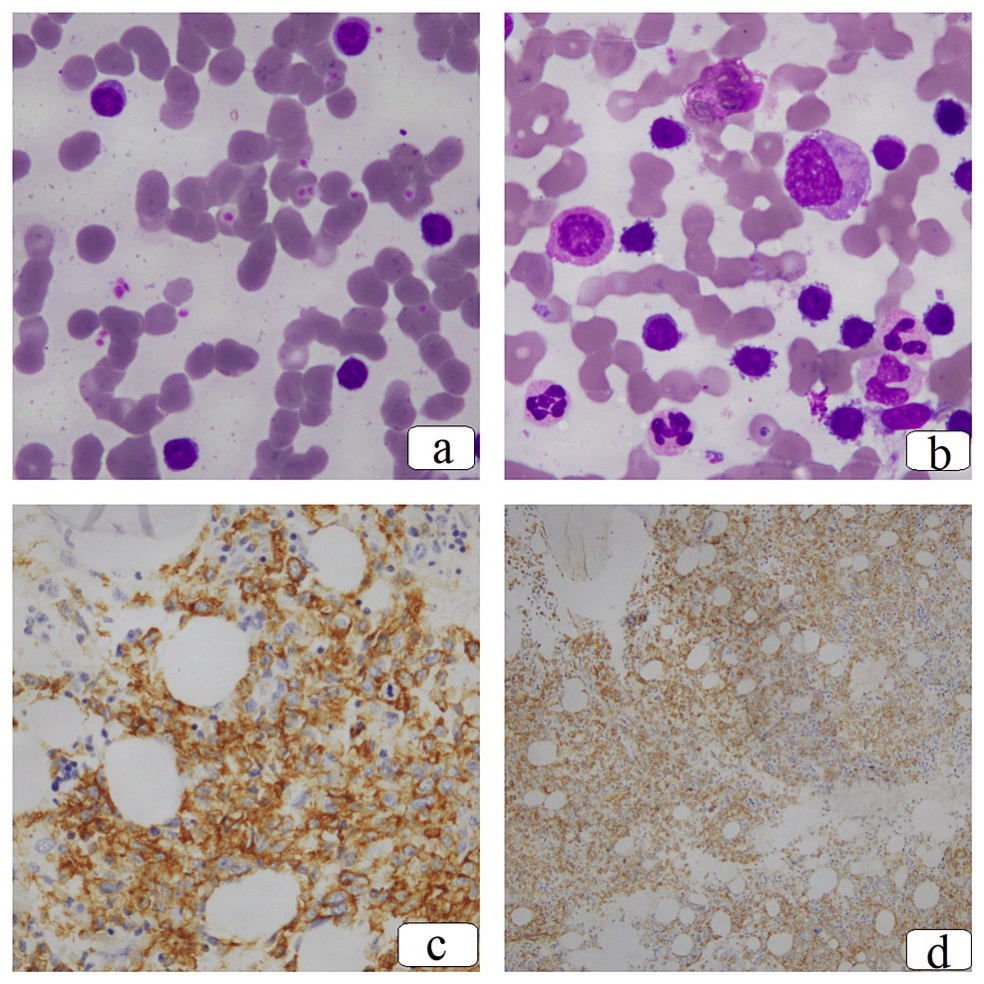
Images of (a) peripheral blood, (b) bone marrow aspiration (Wright-Giemsa x 100), (c) bone marrow biopsy immunohistochemistry (IHC x 40) cyclin D1, (d) and CD20 (before treatment)

A 74-year-old female patient, with a history of ulcerative colitis, was admitted to our center with one month of purpura and bloody stools. On admission, the patient had no fever but had mild anemia, small inguinal lymphadenopathy, and mild splenomegaly. Complete blood count (CBC) showed anemia with 10.5 g/dL of hemoglobin, normal platelet count (152 × 10^3/mL), and elevated white blood cell (WBC) count at 37.7 × 10^3/mL (52% of which was lymphocytes population). Peripheral blood smear imprinted abnormal lymphocytosis with hair-like cytoplasm. A bone marrow biopsy specimen revealed diffuse infiltration of CD20-positive lymphocytes but no lymphoid follicular structure (Figure 1).

**Figure 2:**
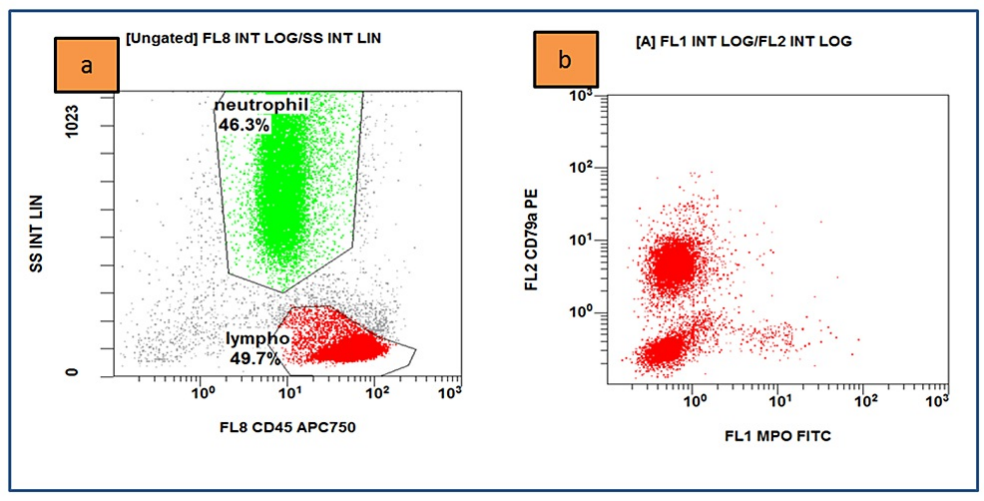
Flow cytometry results on bone marrow sample (a) Lympho cells (red dots) account for 49.7% and are positive for CD45; (b) positive for CD79a (Navios EX, Beckman Coulter, Brea, CA).

**Figure 3:**
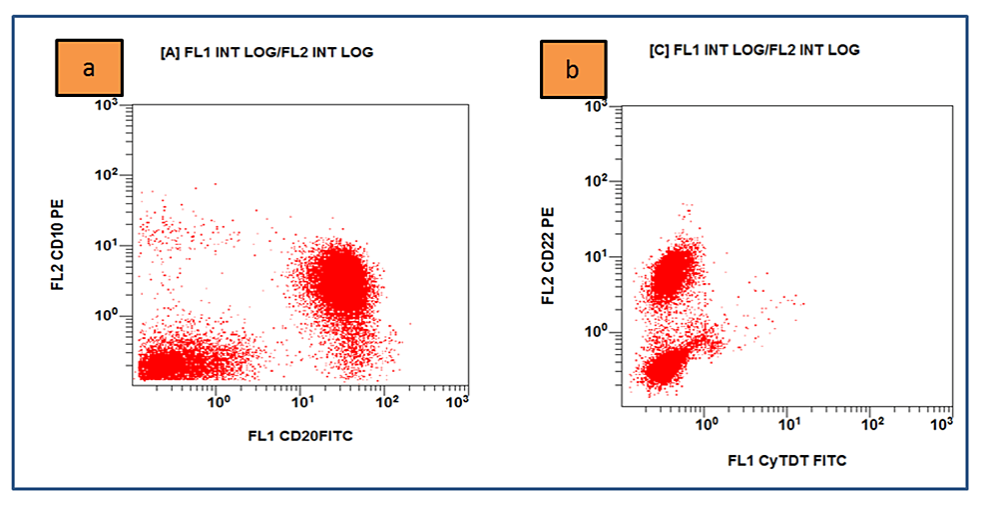
Flow cytometry results on bone marrow sample (a) Lympho cells (red dots) are positive for CD20 and CD10; (b) positive for CD22 and negative for terminal deoxynucleotidyl transferase (Navios EX, Beckman Coulter, Brea, CA).

**Figure 4:**
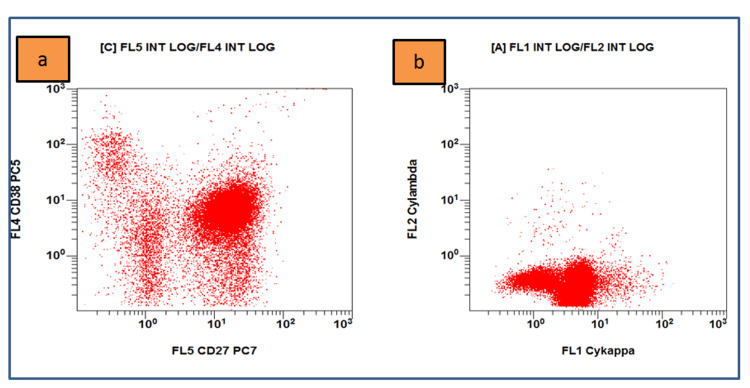
Flow cytometry results on bone marrow sample (a) Lympho cells (red dots) are positive for CD27 and CD38; (b) positive for cyKappa and negative for cyLambda (Navios EX, Beckman Coulter, Brea, CA).

Flow cytometry on bone marrow samples was performed and displayed an increased percentage of B-lineage lymphocytes that were positive for CD19, kappa light chain, CD20, CD22, FMC7, CD79a, CD27, CD11c, CD25, CD10, and CD38 and did not express lambda light chain, CD3, CD4, CD5, CD8, CD23, and CD138 (Figures 2-4). The karyotype test was normal with 46XX. Serum immunoassay showed an increase in IgG level with 2997 U/ml and a decrease in IgA level by 26 U/ml. Biochemical tests of liver and kidney function were within normal limits.

**Figure 5:**
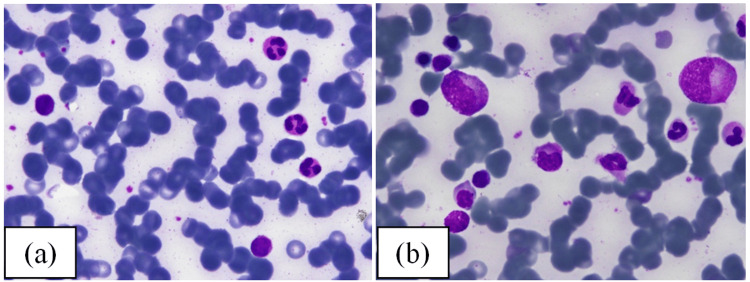
(a) Peripheral blood and (b) bone marrow aspiration (Wright-Giemsa x 100) after two cycles of treatment with RF regimen

The treatment regimen for the patient was rituximab and fludarabine (RF) with an intravenous dose of rituximab (375 mg/m2) and fludarabine (40 mg/m2). After the first RF treatment cycle, the patient was discharged and monitored in the outpatient clinic. At the end of two cycles of RF treatment, the patient had a marked improvement in clinical symptoms, she had no more purpura, and the spleen gradually decreased and was no longer palpable on examination. WBC declined to 11.5 x 10^3/mL, and the lymphocyte percentage was normal at 21% (2.91 x 10^3/mL). Investigation of the bone marrow specimen revealed a normal growth of granulocytes as well as other hematopoietic cells with the normal ratio of myeloid: erythrocytes (Figure 5). At the end of treatment, the patient returned to the hospital and underwent a bone marrow aspiration looking for measurable residual disease (MRD), resulting in 0.8% hairy cells remaining. Currently, the patient is being monitored on an outpatient basis and performed routine laboratory tests to monitor disease progression.

## Discussion

Lymphocytosis, granulocytopenia, and thrombocytopenia are common blood test features in classical HCL. The patient in this report presented with anemia and pathological lymphocytosis. The bone marrow smear imprinted multiple lymphocytes with hair-like cytoplasm, and immunohistochemistry staining of the bone marrow biopsies showed diffuse B-lymphocyte invasion. Immunotyping by flow cytometry helps to differentiate between classical HCL and SMZL, contributing to a definitive diagnosis. The HCL variant is more difficult to distinguish; however, approximately 40% of cases increase the expression of IgG/IgA. In contrast, SMZL cells specifically express IgM with immunoglobulin D and lack IgG or IgA [8]. In our case, the IgG/IgA was 2997/26, as IgG and IgA variations were observed in HCL disease. Therefore, the combination of immunophenotypic analysis of the bone marrow specimen with the clinical and immunoglobulin heavy chain isotype in this patient is consistent with HCL.

Classical HCL is usually negative for CD10 and CD38, about 10-20% are positive for CD10, and 20-30% are positive for CD38 [9,10]. Chen et al. evaluated 35 HCL cases and identified CD10 expression as a marker for B-cell tumors of central follicular origin [11]. CD10 is also considered a marker of poor prognosis in HCL. Classical HCL can express abnormal immune markers such as CD38, which is associated with a shorter period of time to salvage therapy; and in mouse experiments, CD38 can promote survival and adhesion of HCL cells, even shortening the overall survival time to 71 months compared to the normal [12].

In our case, it was found that the patient had strong expression of CD10, CD200, and CD38 markers, which are believed to reduce patient survival time. Differentiation between HCL and other chronic B-cell lymphoid proliferative disorders is essential because hairy cells do not respond well to conventional lymphoma chemotherapy but are remarkably sensitive to purine analogs such as fludarabine. HCL patients are usually treated with monotherapy regimens, but with such poor prognostic markers, we chose a combination regimen of rituximab and fludarabine and it has been shown to be effective. After two months, the patient's CBC parameters improved markedly, the neutrophil increased to normal, and the percentage of hairy lymphocytes reduced. MRD results showed that the percentage of hair cells decreased to less than 1%.

## Conclusions

HCL is a disease that requires a combination of diagnostic options including morphology, histopathology, flow cytometry, and clinical findings to make an accurate diagnosis. Using a list of cell surface immunological markers combined with the clinical and morphological features in this particular case, we had a definite diagnosis of the variant, HCL, although there are a few elements that are not truly standard. We chose the combination regimen of rituximab and fludarabine when abnormal immunophenotypic markers were detected and good treatment results were obtained.
